# Electronic health record closed-loop referral (“eReferral”) to a state tobacco quitline: a retrospective case study of primary care implementation challenges and adaptations

**DOI:** 10.1186/s43058-022-00357-4

**Published:** 2022-10-08

**Authors:** Mark E. Zehner, Julie A. Kirsch, Robert T. Adsit, Allison Gorrilla, Kristine Hayden, Amy Skora, Marika Rosenblum, Timothy B. Baker, Michael C. Fiore, Danielle E. McCarthy

**Affiliations:** 1 Center for Tobacco Research and Intervention, Division of General Internal Medicine, Department of Medicine, University of Wisconsin School of Medicine and Public Health, 1930 Monroe Street, Suite 200, Madison, WI 53711 USA; 2 Department of Family Medicine and Community Health, University of Wisconsin-Madison, 1100 Delaplaine Ct #1896, Madison, WI 53715 USA; 3 Department of Psychology, University of Illinois, Chicago, 1007 W. Harrison St., 1009 BSB, Chicago, IL 60607 USA

**Keywords:** Tobacco, Smoking, Electronic health record, Healthcare systems, Electronic closed-loop referral, Clinical decision support

## Abstract

**Background:**

Health system change can increase the reach of evidence-based smoking cessation treatments. Proactive electronic health record (EHR)-enabled, closed-loop referral (“eReferral”) to state tobacco quitlines increases the rates at which patients who smoke accept cessation treatment. Implementing such system change poses many challenges, however, and adaptations to system contexts are often required, but are understudied. This retrospective case study identified adaptations to eReferral EHR tools and implementation strategies in two healthcare systems.

**Methods:**

In a large clustered randomized controlled trial (C-RCT; NCT02735382) conducted in 2016–2017, 11 primary care clinics in two healthcare systems implemented quitline eReferral, starting with 1 pilot clinic per system followed by 2 phases of implementation (an experimental phase in 5–6 test clinics per system and then a system-wide dissemination phase in both systems). Adaptations were informed by stakeholder input from live trainings, follow-up calls and meetings in the first month after eReferral launch, emails, direct observation by researchers, and clinic staff survey responses. Retrospective, descriptive analysis characterized implementation strategy modifications and adaptations using the Framework for Reporting Adaptations and Modifications to Evidence-based Implementation Strategies (FRAME-IS). A pre- and post-implementation survey assessed staff ratings of eReferral acceptability and implementation barriers and facilitators.

**Findings:**

Major modifications to closed-loop eReferral implementation strategies included aligning the eReferral initiative with other high-priority health system objectives, modifying eReferral user interfaces and training in their use, modifying eReferral workflows and associated training, and maintaining and enhancing interoperability and clinician feedback functions. The two health systems both used Epic EHRs but used different approaches to interfacing with the quitline vendor and integrating eReferral into clinician workflows. Both health systems engaged in iterative refinement of the EHR alert prompting eReferral, the eReferral order, trainings, and workflows. Staff survey comments suggested moderate acceptability of eReferral processes and identified possible targets for future modifications in eReferral, including reducing clinician burden related to EHR documentation and addressing clinicians’ negative beliefs about patient receptivity to cessation treatment.

**Conclusions:**

System-wide implementation of tobacco quitline eReferral in primary care outpatient clinics is feasible but requires extensive coordination across stakeholders, tailoring to local health system EHR configurations, and sensitivity to system- and clinic-specific workflows.

**Trial registration:**

www.clinicaltrials.gov, NCT02735382. Registered on 12 August 2016.

**Supplementary Information:**

The online version contains supplementary material available at 10.1186/s43058-022-00357-4.

Contributions to the literature

- This case study applies a recent adaptation framework to characterize modifications to closed-loop electronic referral to a state tobacco quitline (eReferral) implemented in two health systems.
- We demonstrate that eReferral was an efficient and acceptable way to extend tobacco use treatment in primary care that required tailoring to health system needs and contexts.
- Descriptive analysis identified facilitators and barriers to eReferral implementation that may influence the frequency and quality of referrals.

## Background

Many individuals attempt to quit smoking, but few use the recommended combination of evidence-based counseling and medication [1]. To increase the use of cessation treatment, health systems can adopt system changes to support and encourage clinicians to document smoking status, advise patients to quit, prescribe cessation medications, and refer or connect patients to tobacco cessation treatment (e.g., Ask-Advise-Connect approaches) [2]. Tobacco quitline programs are an effective external source of evidence-based smoking cessation counseling (in all states) and medication (in most states) with few access barriers [3, 4]. However, it is difficult to increase and sustain quitline referral via clinician education and training [5] and quitlines reach only 1–2% of adults who smoke per year [6].

Electronic health record (EHR)-enabled closed-loop referral (eReferral) can facilitate the referral of primary care patients who smoke to a state tobacco quitline [7]. The eReferral workflow prompts clinicians to offer quitline referrals to all patients ready to quit smoking within the next 30 days. The quitline then attempts to reach patients within 24–48 h of receiving an eReferral and later closes the eReferral loop by notifying the clinician of referral outcomes (i.e., whether the patient accepted quitline services and/or received nicotine medication). eReferral appears to reach three times as many primary care patients as quitline fax referral [7–9], with fairly equitable reach across patient subpopulations [8, 9]. However, there are challenges to implementing eReferral [10]. Implementing EHR tools often requires extensive system-specific modifications [11] and engagement of multiple stakeholders [10]. Thus, eReferral often involves multiple rounds of modification and refinement, which can be expensive and time consuming.

Adaptations and refinements are often critical to effective implementation [12] and thereby to intervention reach, effectiveness, and population impact, as specified in the Reach, Effectiveness, Adoption, Implementation, and Maintenance (RE-AIM) framework for intervention planning and evaluation [13]. The process of modifying implementation strategies to support intervention activities in clinical contexts is understudied [14, 15], however, particularly for emerging EHR-guided intervention approaches like Ask-Advise-Connect eReferral [10]. Interventions incorporating EHR-supported implementation may involve adaptations to the basic clinical intervention, modifications to EHR functionalities, and modifications to training processes as well as other implementation strategies. For example, modifications may involve reducing the frequency of EHR prompts to intervene or streamlining EHR navigation (e.g., reducing clicks) to enhance eReferral acceptability and thereby promote its adoption or maintenance and downstream population impact [13, 16, 17]. Such modifications often require the involvement of diverse stakeholders to enhance the acceptability and reduce the burden of an intervention.

The present study examines modifications to EHR tools and implementation strategies supporting tobacco quitline eReferral in Wisconsin, USA, before, during, and after a clustered randomized controlled trial (C-RCT) of eReferral in primary care outpatient clinics across two health systems, System A and System B [9]. Previous research evaluated eReferral versus fax referral (Fax to Quit, or F2Q) to a quitline using the RE-AIM framework [13]. Prior analyses of Wisconsin Tobacco Quit Line (WTQL) referral reach in the first 6 months of implementation indicated that eReferral clinics referred a higher percentage of adult patients who smoke to the WTQL than did F2Q clinics. In System A, the eReferral rate was 17.9% (95% confidence interval [CI], 17.2–18.5%) while the fax referral rate was 3.8% (95% CI, 3.5–4.2%). In System B, the eReferral rate was 18.9% (95% CI, 18.3–19.6%) vs 5.2% (95% CI, 4.9–5.6%) for fax referral [9]. This previously published study on reach and effectiveness did not report on the extensive customization of EHR tools and workflows that occurred in both health systems or on resulting acceptability or perceived eReferral barriers and facilitators among clinic staff. To advance the science of adaptation in the context of implementation [13], the current report assesses the process of modification and adaptation across the deployment of these EHR tools and the acceptability of eReferral among clinic staff.

The present study focuses on modifications to implementation strategies to support eReferral (rather than quitline interventions) that may affect the population health impact of the intervention (e.g., by affecting its adoption, reach, effectiveness, or maintenance), as specified by the RE-AIM framework [13]. The current analyses used the recently proposed Framework for Reporting Adaptations and Modifications to Evidence-based Implementation Strategies (FRAME-IS) [18] to assess modifications to implementation strategies. A pre- and post-intervention survey examined the acceptability of eReferral processes among implementing staff and assessed staff perceptions of barriers and facilitators to eReferral implementation. Two types of modifications were assessed: (1) planned, collaborative adaptations to eReferral implementation strategies intended to enhance RE-AIM dimensions and population impact and (2) unplanned or spontaneous modifications reported by implementing staff in the post-intervention survey.

FRAME-IS guides the systematic documentation of modifications to intervention implementation strategies. This analysis focuses on adaptations and modifications to implementation strategies supporting eReferral. Neither the evidence-based-practice (quitline-provided smoking cessation counseling and nicotine replacement therapy) nor the foundational functions of eReferral (EHR-enabled transfer of patient contact information to a tobacco quitline for proactive telephone outreach to the patient, with return of referral outcomes results to the EHR) changed over the course of the project, i.e., the core components remained unchanged. Instead, modifications and adaptations focused on the mechanisms of eReferral, not treatment content [19], with the aim of improving adoption, reach, implementation, and maintenance [13] of closed-loop, interoperable eReferral to the quitline in adult primary care.

## Methods

### Context

Data for this retrospective case study were derived from a 2-group C-RCT that compared eReferral versus paper faxed referral (F2Q) of adult primary care outpatients to the Wisconsin Tobacco Quit Line (WTQL) [9]. The WTQL offers no-cost proactive phone counseling and 2 weeks of nicotine replacement therapy (NRT) to clients ready to set a target quit date within 30 days. The WTQL offers general information and resources to those not ready to set a quit date within 30 days. This evidence-based treatment did not change during the trial. Primary care clinicians and teams of medical assistants and nurses in 11 primary care clinics in Health System A (5 assigned to eReferral, 6 assigned to fax referral) and 12 clinics in Health System B (6 assigned to eReferral, 6 assigned to fax referral) were trained in WTQL referral using the randomly assigned referral method and implemented that method for at least 6 months. The present case study focuses just on the 11 clinics in the eReferral condition (5 in System A, 6 in System B) and excludes the 12 clinics in the fax referral condition.

Health systems A and B are regional not-for-profit healthcare systems in Wisconsin, and both use the Epic EHR. Details regarding populations served by each health system, clinic eligibility, randomization, masking, workflows, and results are reported in a previously published study [9]. Health system stakeholder support for the adoption and implementation of the C-RCT (and later system-wide dissemination of eReferral) was sought by the research team through collaboration with tobacco cessation treatment champions in both systems.

The research team played the role of a coordinating center and facilitator of eReferral implementation with health system and WTQL stakeholder partners. This coordination involved helping negotiate agreements between health systems and the WTQL for two-way secure data sharing and interoperability. The research team also provided financial resources to cover health system information technology (HIT) costs to ensure that HIT development would be prioritized and would occur in a timely manner to ensure completion of the C-RCT during the grant funding period. Once the HIT teams were assembled, the research team coordinated the development and functional testing of eReferral with the WTQL.

### eReferral workflow

**Fig. 1:**
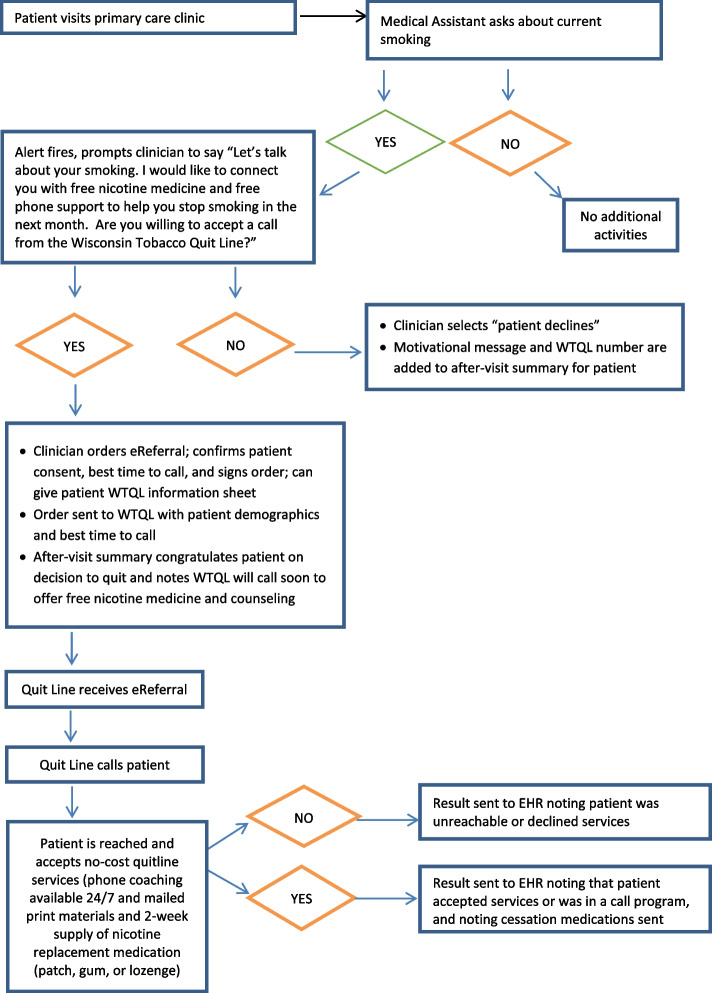
Tobacco quitline eReferral workflow

This case study focuses on WTQL eReferral and not fax referral. Figure 1 shows that the eReferral workflow began with assessment and documentation of smoking status in the EHR, typically by medical assistants or nurses. An EHR eReferral alert appeared when a clinician opened an encounter with a patient who smoked. The alert offered scripted phrases to advise patients to quit smoking, offer WTQL assistance, and assess interest in WTQL support (in that order) and the following response options: “Order” if the patient consented to eReferral, “Patient declines” (System A) or “Refused” (System B) if the patient declined eReferral, or “Defer” if the clinician did not discuss eReferral with the patient (System A only). Clicking “Order” opened a form to verify patient contact information and consent before approving the eReferral order. Clinician approval of the order triggered electronic transmission of the referral to the state quitline (WTQL).

The WTQL proactively called referred patients to offer services and automatically returned results regarding the referral outcome (e.g., patient not reached, patient declined services, patient enrolled in call program, medication dispensed) to the patient’s record in the EHR and the referring provider’s inbox. In System A, any medications dispensed by the WTQL were also automatically added to the patient medication list in the EHR, in addition to being returned to the provider inbox and patient record as part of the referral result report. System B did not adopt automatic updating of patient medication lists.

Although specific settings, processes, and implementation strategies were modified, the core workflow steps and eReferral functions were maintained throughout the study.

### Implementation strategies

**Fig. 2:**
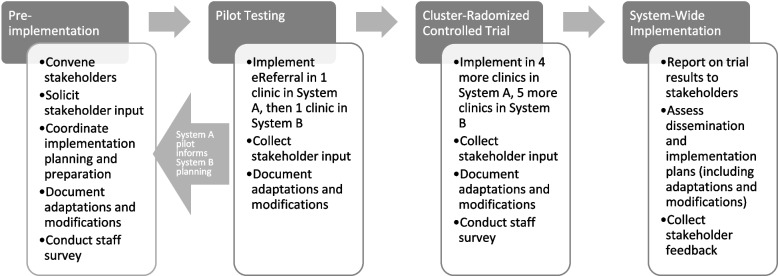
Study activities by implementation phase

Figure 2 depicts the phases of the eReferral implementation and the activities that occurred in each phase. In both health systems, implementation planning engaged leaders and staff in collaborative efforts to tailor Epic-designed EHR tools to the local context and to develop the processes and communication strategies needed to implement and evaluate eReferral. The EHR tools, workflows, and other implementation strategies (e.g., training materials) supporting eReferral were developed collaboratively and refined iteratively with input from healthcare system leaders, clinicians, WTQL personnel, and HIT teams at Epic Systems and the host healthcare systems. Tailoring processes occurred separately in the two health systems, although lessons learned in System A, which launched eReferral first, were shared with stakeholders in System B by the research team (see Fig. 2). In both systems, a 6–8-week period of eReferral pilot testing and adaptation in 1 highly engaged clinic was built into the launch timetable, prior to launching eReferral in 4–5 additional clinics per system.

In all clinics, the eReferral launch was immediately preceded by a 30–60-min live training for clinic staff in the eReferral workflow. A telephone check-in with the clinic manager occurred approximately 1 week later and a second 30–60-min in-person, group feedback session and training refresher with clinic staff occurred approximately 1 month after launch (timing varied across clinics). The WTQL e-mailed monthly summary reports to clinic managers to provide performance feedback and track progress at each clinic.

### Measures

#### Planned modifications and adaptations

Throughout implementation, but particularly during HIT development and pilot testing, the research team facilitated frequent communication among stakeholders to identify challenges to eReferral and to implement relevant adaptations (planned efforts to tailor and improve implementation strategies) [20]. Stakeholders included HIT, health system and clinic staff, and WTQL IT teams. Meeting notes, email communications, training materials, notes on direct observation of workflows by research staff, and stakeholder recollections regarding these challenges and adaptations were systematically collected, reviewed, and categorized for the present qualitative analysis.

#### eReferral acceptability and staff-level modifications

Just before and 6 months after the launch of eReferral in each clinic, the stakeholders who implemented eReferral (clinicians, nurses, medical assistants, and clinic managers) were e-mailed invitations to complete a brief (5-min) anonymous online survey. The survey was preceded by an online consent form. Proceeding to the survey was considered evidence of consent to participate. Fifteen survey items addressed the perceived value of addressing tobacco use, self-efficacy regarding addressing patient tobacco use, and satisfaction with the referral process. The items are shown in Table 1. All were rated on a Likert scale (1 = *strongly disagree*, 7 = *strongly agree*). Quantitative survey responses were treated as cross-sectional because we could not link respondents across waves. Survey responses from Health System A and Health System B were combined for analyses of acceptability and implementer perceptions.

**Table 1:** Clinic staff survey rating means (and standard deviations) by survey wave (pre- vs. 6 months post-implementation)

*Survey items (rated on a scale of 1* = *strongly disagree, to 7* = *strongly agree)*	*Pre (N* = *150)*	*Post (N* = *113)*
1. Addressing tobacco use with patients is very important to me	5.93 (1.28)	6.31 (0.97)*
2. Very few patients will stop using tobacco even with treatment	3.95 (1.60)	4.24 (1.53)
3. My clinic supports me in my attempts to address my patients’ tobacco use	5.93 (1.23)	6.15 (1.01)
4. I have enough time to address my patients’ tobacco use	4.43 (1.76)	4.96 (1.63)*
5. I know what to do to address my patients’ tobacco use	5.37 (1.44)	5.95 (1.06)*
6. I feel that I am part of a good healthcare team that is working well together	6.16 (1.00)	6.23 (1.03)
7. Patients seem to welcome my efforts to address their tobacco use with them	4.33 (1.55)	4.76 (1.44)*
8. The steps I need to take to address my patients’ tobacco use are efficient and well designed	4.72 (1.43)	5.45 (1.22)*
9. The EHR helps me address my patients’ tobacco use	5.07 (1.47)	5.74 (0.95)*
10. I often get feedback on whether my patients got tobacco treatment if they wanted it	3.29 (1.60)	4.12 (1.72)*
11. The Wisconsin Tobacco Quit Line is an effective aid to my patients who want to quit	4.59 (1.49)	5.20 (1.38)*
12. I understand how to refer my patients to the Wisconsin Tobacco Quitline	4.62 (1.94)	6.31 (0.84)*
13. The method to refer patients to the Wisconsin Tobacco Quitline is easy	4.97 (1.54)	5.98 (1.12)*
14. The method to refer patients to the Wisconsin Tobacco Quitline is effective	4.58 (1.49)	5.40 (1.20)*
15. I regularly receive feedback regarding the outcome of the patients I refer to the Wisconsin Tobacco Quitline	2.68 (1.48)	4.21 (1.73)*

^*^Effect of survey wave significant at *p* < .05

In addition, the 6-month post-implementation survey invited implementing staff to identify facilitators and barriers of eReferral and share information about modifications they made to implementation strategies (i.e., what they said to patients in lieu of what was scripted). Open-ended items asked respondents to identify barriers to and facilitators of addressing patients’ tobacco use, and what they typically said to patients to introduce the WTQL.

### Study design and data analyses

This retrospective case study followed the standards for reporting qualitative research, outlined in Table S1 [21]. Retrospective, descriptive analysis characterized implementation strategy modifications identified by any means (e.g., observation, oral or written communication with stakeholders, post-implementation survey responses). The FRAME-IS [18] was applied to all logged adaptations by two authors (JK and DM), using all applicable modules: (1) a description of the treatment and implementation strategies, (2) what was modified, (3) the nature of the modification, (4) the rationale for the modification, (5) when the modification occurred and whether it was planned, (6) who participated in the decision to modify, and (7) how widespread the modification was.

Acceptability and staff perceptions were analyzed via descriptive statistics and ANOVA tests of pre-post differences in the 15 quantitative staff survey items using R 3.6.2. Free responses on the staff survey were treated as qualitative data. Illustrative individual responses and common themes were summarized after independent extraction and discussion by two authors (JK and DM).

## Results

### Modifications and adaptations

Table S2 in the supplementary material lists all major modifications with all core and supplementary FRAME-IS modules [18] for each modification. Table 2 summarizes the nature (module 2) and goals (module 4) of key, planned adaptations logged in Table S2. All modifications were organized under four implementation strategy categories based on post hoc examination of the responses by JK and DM: aligning the eReferral initiative with other high-priority health system objectives, modifying eReferral user interfaces and training in their use, modifying eReferral workflows and associated training, and maintaining and enhancing interoperability and clinician feedback functions.

**Table 2:** Summary of key modifications and adaptations to eReferral strategies before, during, and after eReferral implementation in two healthcare systems

*Summary of proactive, planned modifications (adaptations)*	*Modification goal*
Align eReferral initiative with other high-priority **health system objectives**: •Configure eReferral so it can demonstrate meaningful use of EHR •Align interoperability approach to system HIT resources and infrastructure	Increase adoption and sustainment system-wide, align with sociopolitical-level mandates
Engage health system stakeholders (clinicians, HIT developers and trainers, quality improvement leaders, smoking cessation champions, clinic managers) to tweak and refine eReferral **user displays** and training in their use: •Make EHR eReferral alert format highly salient •Remove “hard-stop” and reduce frequent alert firing that burdens clinicians •Prompt clinicians to offer assistance in quitting smoking before assessing patient readiness to quit •Prepopulate as many fields as possible to reduce data entry burden •Remove system defaults that increase burden without adding needed functionality •Add deferral option for clinicians who routinely pre-chart	Enhance adoption, fidelity, acceptability, and sustainability among clinicians (and downstream reach among patients) system-wide
Engage health system stakeholders (clinicians, HIT developers and trainers, quality improvement leaders, smoking cessation champions, clinic managers) to tweak and refine eReferral **workflows** and associated training: •Prompt eReferral in all face-to-face encounters with all primary care clinicians (including physicians and advanced practice providers) •Train medical assistants who conduct rooming activities in the importance of logging out of encounters (vs. securing login session) to facilitate clinician eReferral workflow •Automate incorporation of quitline information in after-visit summaries for all patients for whom the EHR alert fired (whether eReferred during the visit or not) so this happens consistently and without additional clinician data entry	Enhance adoption, fidelity, acceptability, and sustainability among clinicians’ reach among patients system-wide
Maintain and enhance **interoperability** and **clinician feedback** functions: •Monitor and maintain interoperability functioning, especially after system updates •Modify returned eReferral results so they cover all possible outcomes and are clear to clinicians making eReferrals •Adapt QuitLine standards to increase yield from eReferrals	Increase adoption, acceptability, sustainability, and/or reach

#### Aligning the eReferral initiative with other high-priority healthcare system objectives

Adaptations to align eReferral to healthcare system goals and policies began in the health system recruitment process and continued throughout implementation planning and pilot testing. During the planning phase in System B, stakeholders in HIT alerted the research and implementation planning team to potential misalignment between the proposed interface mechanism with the WTQL and System B interoperability capabilities and preferences. Based on this, the nature of the interface supporting interoperability was adapted to the System B HIT context, while preserving core interoperability functions and HIPAA compliance.

In addition, during the pilot testing phase in System A, clinical and HIT stakeholders suggested ways to redesign the eReferral alert so it would meet the criteria for Meaningful Use of the EHR [22] by documenting that clinicians advised cessation and offered assistance to patients, even if patients declined eReferral. This adaptation was adopted by System B, as well.

#### Modifying eReferral user interfaces and training in their use

Extensive stakeholder input was gathered and used to adapt the eReferral alert and order in the EHR throughout the planning, piloting, and implementation study phases (see Table S2). The research team served as the hub linking programmers across Epic, healthcare systems, and the WTQL to achieve customization, configuration, testing, and refinement of eReferral tools and interfaces. Training in the use of the eReferral alert and order was also modified based on piloting and mid-implementation feedback. As shown in Tables S2 and 2, many aspects of eReferral were adapted to and by systems, with the goals of increasing both salience and ease and efficiency of use, and thereby enhancing adoption, fidelity, acceptability, and sustainability among clinicians and increasing eReferral reach among patients. Key modifications included changes in alert design and content; firing rules (e.g., whether the alert compelled a response before closing an encounter in the EHR, known as a “hard-stop”); order set defaults/prepopulated fields; and removal of burdensome traditional referral follow-up activities (e.g., assessment of patient cognitive and motor ability to follow-through with external referrals).

Variation in clinician charting practices also necessitated adaptation of the eReferral alert. Some clinicians routinely started documentation in encounters before seeing the patient (i.e., engaged in “pre-charting”), which caused the alert to present prematurely. In System A, where a “hard-stop” firing rule was in place, clinicians selected “patient declined,” without ever engaging the patient, to conclude their pre-charting (as they had to select a response in the alert to close the chart). Selecting “patient declined” during pre-charting suppressed the appearance of the alert during the patient-facing part of the encounter and reduced opportunities for clinicians to discuss eReferral with patients. To accommodate pre-charting, a “Defer” option was added to the alert. A “Defer” option allowed clinicians to satisfy the “hard-stop” alert and close the chart after pre-charting, while also allowing the alert to fire again when the encounter was reopened when the clinician was with the patient. Clinic and system stakeholders in System A suggested and implemented this solution and trained clinic leaders and clinicians in this added functionality in the alert. Health System B, on the other hand, did not implement a “hard-stop” rule and did not need to add a “Defer” option to facilitate chart closure during pre-charting and so offered only “Order” and “Refused” options in the alert.

#### Modifying eReferral workflows and associated training

To affect clinical care, EHR tools like eReferral must be integrated into sustainable workflows. In both systems, prior to WTQL eReferral implementation, assessing and addressing tobacco use was often assigned to medical assistants or nurses. In this study, eReferral was designed to involve clinicians/providers because the literature points to the efficacy of brief clinician counseling regarding quitting [23]. In addition, quitline eReferral was intended to be a treatment extender, rather than a substitute for clinician intervention. As such, clinical informatics and HIT stakeholders helped iteratively refine criteria for eReferral alert firing so that it appeared only during clinician-facing encounters that afforded opportunities to address tobacco use (e.g., not just blood pressure checks or orders-only encounters), and so that it appeared for all clinicians able to offer cessation counseling and pharmacotherapy (including advanced practice providers in addition to physicians).

Stakeholder input also led to the inclusion of automated messages regarding quitline services to after-visit summaries provided to all patients for whom the eReferral alert fired. These messages were tailored to clinician eReferral alert response. For patients who accepted eReferral, a congratulatory message encouraged them to accept the call from the WTQL that would come in a few days. For all others (those who declined eReferral and those not offered it), the visit summary described WTQL services and provided the toll-free quitline number.

Stakeholder input also identified why some clinicians addressed the eReferral alert at low rates. When medical assistants or other staff who conducted rooming activities secured the EHR session at the workstation in the clinic room with the encounter open (rather than logging out fully), this altered the appearance of the eReferral alert making it less prominent for clinicians. To address this, clinic managers provided additional training to clinic staff who conducted rooming activities to log out fully of the workstation (rather than just secure it).

Post-implementation staff surveys indicated that some primary care teams continued to shift smoking treatment activities to medical assistants or nurses even after adopting eReferral. After data collection ended, one system shifted primary responsibility for eReferral back to medical assistants (with clinician approval of eReferral orders prepared by medical assistants) while the other retained the clinician-centered workflow when eReferral was adopted as the standard of care system-wide.

#### Maintaining and enhancing interoperability and clinician feedback functions

Interfaces between the WTQL and the EHR were disrupted by updates and changes at either entity (e.g., when security certificates were updated). In pilot and mid-implementation phases, clinicians and clinic managers reported concerns regarding the length of time it took to receive patient outcomes from the WTQL. Tweaking of interoperability and WTQL feedback reports was required to ensure that feedback provided to referring clinicians was timely, complete, and clear (see Tables S2 and 2). In addition, the WTQL modified its eReferral response timeline (reducing the window for making a first outbound call to eReferred patients from 48 to 24 h) to increase the rate at which eReferred patients were successfully reached by WTQL quit coaches. In addition, in System A, stakeholders indicated that they wanted interoperability to extend to patient medication lists. To accommodate this request, any medications dispensed to patients by the WTQL were automatically added to their active medication lists. System B, on the other hand, did not permit automatic updating of patient medication lists with medications provided outside the system, but still allowed automatic posting of results in the referrals section of patient charts, and routing of these results to clinician inboxes. Clinicians and clinic staff reported to the eReferral team when eReferral results were missing, unclear, or incomplete. One clinician noted on the post-implementation survey that they stopped eReferring patients because so many patients had results indicating they were unreachable. These results suggest that clinicians and clinic teams watched for and looked at eReferral result reports from the WTQL and that closing the eReferral loop is important to primary care teams.

### eReferral acceptability and staff-level modifications

Post-implementation survey results suggest possible targets for future modifications in eReferral implementation strategies. Table 1 reports means and standard deviations of quantitative survey responses among staff at eReferral clinics both just before and 6 months after eReferral launch, collapsed across clinic and health system. Ratings of the importance of addressing tobacco use, knowledge of ways to address patient tobacco use, clinic support for tobacco use intervention, and team functioning were high (near the top of the 7-point rating scale) at both waves. Post-eReferral implementation, mean ratings on the items assessing agreement that tobacco treatment workflows and quitline eReferral procedures, in particular, are clear, easy, efficient, effective, and helpful were all above 5 on the 7-point scale, signaling agreement with these positive statements. Ratings at both time points also reflected some concerns. On average, respondents agreed slightly that they did not have enough time to address patient tobacco use and the mean score on items assessing patient receptivity and responsivity to treatment were neutral (neither agree nor disagree). Mean responses were also neutral for receiving feedback on tobacco treatment (and quitline) outcomes. Although many ratings improved significantly across waves, especially in terms of the EHR helping address tobacco use, WTQL referral ease, and receipt of feedback regarding WTQL referral outcomes, it is not clear that the improvements were due to changes in respondents’ views or differences in the samples who completed the surveys.

In addition to providing ratings, 106 of the 113 (93.8%) post-implementation survey respondents completed open-ended survey questions about their perceptions of WTQL referral facilitators and barriers. Many responses identified lack of time or time pressure as a barrier to addressing tobacco use well. One respondent wrote “With everything else that is being thrown at primary care, I find I have less and less time to address even important things like smoking cessation at office visits.” Another described the following barrier: “Increasing demands on time in regard to satisfying electronic medical records and organizational requirements for best practice protocol. This results in much less time with patients and much more time in front of the computer.”

Several clinicians, medical assistants, and nurses cited patient lack of interest in quitting and/or resistance to treatment as eReferral barriers. For example, one medical assistant noted: “… most of all it is the willingness of the patient. Often when they say they are interested they lose all of their interest when walking out the door.” Some clinicians reported modifying the eReferral offer language by first assessing patients’ readiness to quit before referring to the quitline. Clinician and staff respondents in eReferral clinics mentioned barriers related to low WTQL connection rates. One noted: “Referral process to the tobacco quit line was okay, but it seemed like the majority of time they were unable to reach my patients for some reason. It ended up being a waste of time, so I stopped doing it.”

Many open-ended responses, especially from medical assistants, endorsed the effectiveness of clinic eReferral protocols in engaging patients in tobacco cessation treatment, but some responses suggested that EHR and workflow steps could be more efficient. For example, one respondent noted that the BPA alert fires at inopportune times in their workflow: “The BPA fires before even entering the room and then again as I try to exit the room, so often times it is an after-thought.” Another respondent wrote: “It would be nice to have better flow within our EHR. Currently we have the tobacco quitline initially, then a social history section in one place, best practice advisories in another place, and no clear way of pulling documentation into our notes.”

In addition, consistent with stakeholder input before and during implementation (Tables S2 and 2), many clinic staff in eReferral clinics noted that task sharing and workflows could be improved by using a team approach (“provider, nurse, coach, etc.”) or having nurses provide cessation counseling and education. Some nurses noted, though, that inadequate communication with clinicians and insufficient clinician support and trust for nurses or medical assistants to offer smoking treatment were barriers to tobacco treatment. Some reported, too, that patients want to receive quitting advice from their physicians.

## Discussion

Implementation of eReferral to a state tobacco quitline in primary care clinics in two regional healthcare systems required extensive stakeholder engagement in implementation monitoring and adaptation and achieved moderate acceptability ratings among implementing clinic teams. Customizing EHR tools, workflows, and training for healthcare systems requires engaging diverse stakeholders at multiple stages of planning, piloting, and implementation. Key implementation strategies modified to enhance eReferral implementation included aligning eReferral with health system priorities; refining EHR eReferral displays, workflows, and training; and maintaining interoperability and closed-loop eReferral functionality. Surveys of clinic staff indicated that the adapted approach had moderate acceptability in terms of clarity, ease, efficiency, and effectiveness. Survey responses also suggested that limited time to address tobacco use and lack of positive feedback regarding client acceptance of quitline services are barriers to eReferral.

Applying the FRAME-IS framework to the analysis of meeting notes, email chains, training documents, and open-ended clinician and clinic staff survey responses regarding eReferral implementation identified several planned, system-wide adaptations. These adaptations were purposive to increase adoption, feasibility, acceptability, and sustainment of tobacco quitline eReferral in adult primary care, and/or to increase reach among patients so more were effectively connected with evidence-base quitline interventions. Diverse healthcare system stakeholders (clinicians, clinic managers, medical assistants, system tobacco cessation champions, health informatics trainers, HIT designers) helped to identify challenges and develop adaptations to address these challenges. Although the research team played a coordinating role in this process, most of the problems and solutions were generated by healthcare system personnel.

In addition to planned, system-wide adaptations, other, more local modifications were identified in the present analysis. Check-ins with front-line clinic staff during site visits and refresher trainings, and calls with clinic managers mid-implementation, helped to identify specific workflow practices that posed challenges (e.g., pre-charting, securing vs. logging out of encounters after rooming). Some of these challenges were addressed by system-wide solutions (e.g., adding a “defer” option to the eReferral alert for clinicians who see the alert during pre-charting in System A), while others involved more local solutions (e.g., training medical assistants and nurses who complete rooming activities to log out of the EHR completely to enhance eReferral alert effectiveness as a cue to clinicians). These local modifications and solutions suggest that even for presumptively straightforward EHR-based strategies, stakeholder input on specific clinic practices and contexts should be collected during implementation planning.

Post-implementation staff survey responses identified moderate levels of agreement that eReferral is clear, easy, efficient, and effective. Survey responses identified barriers to eReferral, including limited time and insufficient or negative feedback about patient results. Open-ended responses among some staff underscored concerns about eReferral workflow efficiency (e.g., the alert appearing at inopportune times) and effectiveness (e.g., few eReferred patients actually receive quitline services). Open-ended responses also captured variability in the way eReferral was implemented at the individual staff level (e.g., what clinicians actually say to patients vs. what is scripted in the eReferral alert). Assessing the implementation perspectives and practices of a broad range of clinicians and staff earlier in implementation may provide a rich source of possible adaptation targets (e.g., reducing alert fatigue) [16] and strategies (e.g., developing a workflow that permits but does not require task sharing).

Survey respondents identified challenges to addressing tobacco use in primary care that are consistent with other evidence on barriers to and facilitators of smoking treatment implementation in healthcare [15, 16, 24–26]. For example, survey responses indicated no improvement from pre- to post-implementation in the belief that patients would stop using tobacco with treatment. Open-ended responses detected low levels of confidence in patient receptivity to smoking cessation advice and assistance. While most patients who smoke report wanting to quit smoking [1], this is likely an inaccurate index of their willingness to begin smoking cessation treatment at a particular encounter. It may be important that clinic staff learn that offering smoking treatment increases rather than decreases patient satisfaction [27]. Negative beliefs may inhibit staff engagement in eReferral and may be strengthened by feedback that patients were not reached or enrolled in quitline services.

### Limitations

The present results should be interpreted with the following limitations in mind. First, our methods of assessing adaptations may have captured more centralized, planned adaptations than local and/or unplanned modifications. As such, our modification list is likely not exhaustive or fully representative of modifications in quitline eReferral. Second, staff survey responses were modest overall and lower at the 6-month post-implementation wave than at the pre-implementation wave. As such, the comparability of samples across waves is unclear and the representativeness of the respondent sample is unknown. Because these surveys were anonymous and treated as cross-sectional rather than longitudinal, we do not know if these changes across the waves reflect within-person changes or sampling differences (e.g., engaging respondents with more knowledge, confidence, and positive perceptions of the WTQL post- vs. pre-implementation). The primary purpose of the quantitative survey analyses was to assess the acceptability of eReferral processes after its implementation, rather than change in attitudes over time. The pre-implementation ratings are reported to provide context rather than comparison with post-implementation and adaptation and results. Third, adaptations were intended to improve the adoption, feasibility, acceptability, and reach of eReferral. Because this was a retrospective case study, we were not able to directly assess the impacts of specific adaptations on these outcomes. In future research, proactive application of other frameworks, such as the Model for Adaptation Design and Impact (MADI) [28], can guide adaptation planning and assessment prior to and during implementation, and evaluation of adaptation effects on implementation outcomes. 

### Conclusions

Widely adopted EHR platforms, including Epic, offer tools to support eReferral to low-barrier, no-cost evidence-based smoking cessation treatment offered at no cost to health systems or patients by state tobacco quitlines. Engaging health system and quitline stakeholders in planned, centralized adaptation of implementation strategies may help to align quitline eReferral with broader priorities and objectives, engage stakeholders in interface and workflow refinement, and maintain interoperability and feedback loops in ways that are acceptable and sustainable in adult primary care settings.

## Supplementary Information

**Additional file 1: Table S1.** Standards for Reporting Qualitative Research (SRQR). **Table S2.** Modifications and adaptations to eReferral strategies before, during, and after eReferral implementation in two healthcare systems (HS).

## Data Availability

The datasets generated are available from the corresponding author on reasonable request.

## Abbreviations

EHR: Electronic health record
eReferral: Electronic health record enabled closed-loop referral
FRAME-IS: Framework for Reporting Adaptations and Modifications to Evidence-based Implementation Strategies
F2Q: Fax to quit
RE-AIM: Reach, Effectiveness, Adaptation, Implementation, and Maintenance framework
