# Supplementary material for: Electronic health record closed-loop referral (“eReferral”) to a state tobacco quitline: a retrospective case study of primary care implementation challenges and adaptations
Source: Implement Sci Commun. 2022 Oct 8;3:107. doi: 10.1186/s43058-022-00357-4 (PMC9548147; doi:10.1186/s43058-022-00357-4)
Supplement: Supplementary file 1 — Additional file 1: Table S1. Standards for Reporting Qualitative Research (SRQR). Table S2. Modifications and adaptations to eReferral strategies before, during, and after eReferral implementation in two healthcare systems (HS). [file 43058_2022_357_MOESM1_ESM.docx]

| Table S1. Standards for Reporting Qualitative Research (SRQR) | | |
| --- | --- | --- |
|  |  | **Page/line no(s).** |
| **Title and abstract** | |  |
|  | **Title** - Concise description of the nature and topic of the study Identifying the study as qualitative or indicating the approach (e.g., ethnography, grounded theory) or data collection methods (e.g., interview, focus group) is recommended | 1 |
|  | **Abstract** - Summary of key elements of the study using the abstract format of the intended publication; typically includes background, purpose, methods, results, and conclusions | 2 |
|  |  |  |
| **Introduction** | |  |
|  | **Problem formulation** - Description and significance of the problem/phenomenon studied; review of relevant theory and empirical work; problem statement | 4-6 |
|  | **Purpose or research questio**n - Purpose of the study and specific objectives or questions | 6 |
|  |  |  |
| **Methods** | |  |
|  | **Qualitative approach and research paradigm** - Qualitative approach (e.g., ethnography, grounded theory, case study, phenomenology, narrative research) and guiding theory if appropriate; identifying the research paradigm (e.g., postpositivist, constructivist/ interpretivist) is also recommended; rationale** | 11 |
|  | **Researcher characteristics and reflexivity** - Researchers’ characteristics that may influence the research, including personal attributes, qualifications/experience, relationship with participants, assumptions, and/or presuppositions; potential or actual interaction between researchers’ characteristics and the research questions, approach, methods, results, and/or transferability | NA |
|  | **Context** - Setting/site and salient contextual factors; rationale** | 7-9 |
|  | **Sampling strategy** - How and why research participants, documents, or events were selected; criteria for deciding when no further sampling was necessary (e.g., sampling saturation); rationale** | NA |
|  | **Ethical issues pertaining to human subjects** - Documentation of approval by an appropriate ethics review board and participant consent, or explanation for lack thereof; other confidentiality and data security issues | 10 |
|  | **Data collection methods** - Types of data collected; details of data collection procedures including (as appropriate) start and stop dates of data collection and analysis, iterative process, triangulation of sources/methods, and modification of procedures in response to evolving study findings; rationale** | 9-11 |
|  | **Data collection instruments and technologies** - Description of instruments (e.g., interview guides, questionnaires) and devices (e.g., audio recorders) used for data collection; if/how the instrument(s) changed over the course of the study | 10-11 |
|  | **Units of study** - Number and relevant characteristics of participants, documents, or events included in the study; level of participation (could be reported in results) | 16, 29 |
|  | **Data processing** - Methods for processing data prior to and during analysis, including transcription, data entry, data management and security, verification of data integrity, data coding, and anonymization/de-identification of excerpts | 10-11 |
|  | **Data analysis** - Process by which inferences, themes, etc., were identified and developed, including the researchers involved in data analysis; usually references a specific paradigm or approach; rationale** | 11 |
|  | **Techniques to enhance trustworthiness** - Techniques to enhance trustworthiness and credibility of data analysis (e.g., member checking, audit trail, triangulation); rationale* | 10-11 |
|  |  |  |
| **Results/findings** | |  |
|  | **Synthesis and interpretation** - Main findings (e.g., interpretations, inferences, and themes); might include development of a theory or model, or integration with prior research or theory | 11-16 |
|  | **Links to empirical data** - Evidence (e.g., quotes, field notes, text excerpts, photographs) to substantiate analytic findings | 16-18 |
|  |  |  |
| **Discussion** | |  |
|  | **Integration with prior work, implications, transferability, and contribution(s) to the field -** Short summary of main findings; explanation of how findings and conclusions connect to, support, elaborate on, or challenge conclusions of earlier scholarship; discussion of scope of application/generalizability; identification of unique contribution(s) to scholarship in a discipline or field | 18-20 |
|  | **Limitations** - Trustworthiness and limitations of findings | 21 |
|  |  |  |
| **Other** | |  |
|  | **Conflicts of interest** - Potential sources of influence or perceived influence on study conduct and conclusions; how these were managed | 22-23 |
|  | **Funding** - Sources of funding and other support; role of funders in data collection, interpretation, and reporting | 22-23 |
|  | Note. *The rationale should briefly discuss the justification for choosing that theory, approach, method, or technique rather than other options available, the assumptions and limitations implicit in those choices, and how those choices influence study conclusions and transferability. As appropriate, the rationale for several items might be discussed together. |  |

Table S2. Modifications and adaptations to eReferral strategies before, during, and after eReferral implementation in two healthcare systems (HS).

| *Module 1: Modifications* | *Module 2: What is modified?* | *Module 3: Nature of the modification?* | *Module 4: What is the goal?* | *Module 5: When is the modification made* | *Module 6: Who participates in the decision to modify?* | *Module 7:*  *How widespread is the modification?* |
| --- | --- | --- | --- | --- | --- | --- |
| ***Align eReferral initiative with other high-priority health system objectives*** | | | | | | |
| Aligned eReferral documentation activities with Meaningful Use-Health Maintenance Criteria so eReferral activities advance health system objectives | **Evaluation:** Configuration of EHR alert to meet multiple objectives including meaningful use evaluation | Tweaking; Core functions preserved | Increase adoption and sustainment, align with sociopolitical-level mandates | Pilot; planned; reactive to HS input | HS clinicians, leaders, HIT | HS-wide (within system) |
| Aligned nature of secure, HIPAA-compliant interface between HS and QL in HS B (https with SSL) vs. A (VPN) with available technological and support resources and system preferences and capabilities at HS B | **Context:** Modification to interoperability interfaces | Tailoring; Core functions preserved | Expedite development, align with system interface capabilities to increase adoption and sustainment at the system level | Pre; planned; reactive to HIT input | HIT, QL, Epic | HS-wide |
| ***Configure electronic health record (EHR) tools to tweak and refine eReferral user displays and training in their use*** | | | | | | |
| Alert appearance and placement changed to be more eye-catching to clinicians using a pop-up window in HS A and brightly colored graphic alert in the alert tab for encounters in HS B | **Format:** EHR alert format modified | Change in materials; Core functions preserved | Increase clinician engagement in QL eReferral by making alert more salient to clinicians | Pre; Planned; reactive to HIT input | HIT | HS-wide |
| *Module 1: Modifications* | *Module 2: What is modified?* | *Module 3: Nature of the modification?* | *Module 4: What is the goal?* | *Module 5: When is the modification made* | *Module 6: Who participates in the decision to modify?* | *Module 7:*  *How widespread is the modification?* |
| ***Configure electronic health record (EHR) tools to tweak and refine eReferral user displays and training in their use, cont…*** | | | | | | |
| Removed eReferral “hard-stop” requiring clinician response to EHR alert for eReferral to reduce burden on clinicians | **Format and Training**: Removing recommended “hard-stop” | Loosening structure; Core functions preserved | Increase acceptability and sustainment; reduce clinician burden | Pre; planned; reactive to HS input | HS leaders, clinicians, HIT | HS-wide in HS B only |
| Alter clinician-facing alert language modified to assess readiness after offering QL assistance, to enhance reach among those who may lack confidence regarding quitting without treatment or have concerns about treatment affordability or access | **Content and Training**: Cue clinicians to describe QL services before assessing interest in quitting and eReferral | Change in materials; Core functions preserved | Increase reach and fidelity | Pre; planned; proactive | UW-CTRI outreach, HS clinical informaticists, HIT | HS-wide |
| Reduced number of times eReferral alert will fire in a single day by increasing suppression window when patient declines from 2 to 24 hours | **Format:** Reducing frequency of intervention offers | Reducing frequency of intervention offers; Core functions preserved (eReferral alert still fires daily) | Increase acceptability by reducing patient and clinician burden | Pilot; planned; reactive to HS input | HS clinicians, HIT | HS-wide |
| Changed eReferral alert response options to accommodate clinician precharting by adding “defer” option that would not suppress alert during subsequent encounter | **Format and Training:** Modified clinician alert and training | Tweaking; Core functions preserved | Increase reach and acceptability | Post; planned; reactive to HS input | HS clinicians; HIT | HS-wide |
| *Module 1: Modifications* | *Module 2: What is modified?* | *Module 3: Nature of the modification?* | *Module 4: What is the goal?* | *Module 5: When is the modification made* | *Module 6: Who participates in the decision to modify?* | *Module 7:*  *How widespread is the modification?* |
| ***Tweak and refine eReferral workflows and associated training*** | | | | | | |
| Limited eReferral alert firing to face-to-face encounters with clinicians who can counsel and prescribe medications; Suppressed alert for encounters just for refills, telephone, email, letters, orders, documentation | **Setting:** Limiting implementation to encounters with clinicians | Tweaking; Core functions preserved | Increase clinical effectiveness by offering eReferral with clinician advice and assistance | Pre, Pilot, and Post; planned; reactive to HS input | UW-CTRI outreach, HS clinical informaticists, HIT | HS-wide |
| Set rules about who sees the alert to include RNs, NPs, PAs, DOs, and residents providing face-to-face encounters, not just attending MDs | **Personnel:** Engage all primary care clinicians in eReferral implementation | Tweaking; Core functions preserved | Increase adoption and reach | Pre and Pilot; planned; proactive | HS clinicians, HIT | HS-wide |
| Medical assistants shared the tasks of advising patients to quit, offering QL referral, and assessing readiness to quit to reduce clinician burden | **Personnel:** Engage all primary care clinicians in eReferral implementation | Substituting; Core functions (clinician advice) changed | Increase reach and reduce clinician burden | Pre, Pilot, Mid, Post; unplanned | HS primary care teams | Specific primary care teams Mid; HS A shifted eReferral to MAs Post |
| Removed workflow defaults: 1) requiring diagnosis for order; 2) printing orders; 3) cognitive and functional assessments for external referrals to reduce burden on primary care staff | **Context:** Removing elements added by HIT defaults | Tweaking; Core functions preserved | Increase acceptability | Pilot, Mid; planned, reactive to HS input | HS clinicians and clinic staff, HIT | HS-wide |
| *Module 1: Modifications* | *Module 2: What is modified?* | *Module 3: Nature of the modification?* | *Module 4: What is the goal?* | *Module 5: When is the modification made* | *Module 6: Who participates in the decision to modify?* | *Module 7:*  *How widespread is the modification?* |
| ***Tweak and refine eReferral workflows and associated training, cont…*** | | | | | | |
| Trained clinic staff conducting rooming (MAs or nurses) to log out rather than secure EHR when leaving the room, | **Training:** Emphasized the consequences of securing (vs. logging out) in terms of what the clinician sees | Tweaking; Core functions preserved | Improve fidelity and reach | Mid; planned, proactive | HS MAs, nurses, EHR trainers | Clinic-specific |
| Filling in eReferral order fields was burdensome, so prepopulated eReferral order fields to minimize clinician data entry; Set default billing and diagnostic codes for all eReferral orders, pulled in patient phone numbers from registration data, and set “any time” as the preferred time for calls | **Context and Training:** Reduced data entry demands on clinicians by prepopulating required fields | Tweaking; Core functions preserved | Improve clinician adoption and acceptability; decrease costs and clinician burden | Pre, Pilot; Planned, proactive | HS clinicians, HIT | HS-wide |
| To enhance reach, added congratulatory reminder that QL will call to visit summary for eReferred patients; added motivational message with QL number for those not eReferred | **Content and Format:** Automated text appended to patient visit summary for all patients for whom the alert fired | Change in materials, adding elements; Core functions preserved | Increase reach | Pre; planned; reactive to HS input | HS clinician stakeholders, HIT | HS-wide |
| *Module 1: Modifications* | *Module 2: What is modified?* | *Module 3: Nature of the modification?* | *Module 4: What is the goal?* | *Module 5: When is the modification made* | *Module 6: Who participates in the decision to modify?* | *Module 7:*  *How widespread is the modification?* |
| ***Tweak and refine eReferral workflows and associated training, cont…*** | | | | | | |
| Clinician survey responses indicated that some clinicians modified eReferral offer language (e.g., to first assess readiness to quit before offering assistance, to emphasize that the QL is for people serious about quitting) | **Content:** Clinicians modified patient messaging and invitations regarding eReferral | Drift; unknown if core functions preserved | Unknown | Mid; unplanned | HS clinicians | Clinician-specific |
| ***Maintain and enhance interoperability and clinician feedback functions*** | | | | | | |
| Improved and maintained connectivity between HS and QL vendor to foster clear feedback about eReferral results | **Context:** Maintaining connectivity and feedback mechanisms required ongoing monitoring and maintenance | Tweaking; Core functions preserved | Increase acceptability and sustainability | Mid; planned, reactive to connection problems | HS clinicians, HIT, QL | HS-wide |
| Enhance utility of feedback by automatically adding nicotine medication provided by the WTQL to patient medication lists in the EHR | **Content:** Medication information was returned in a more useful format for clinic teams | Tweaking; Enhancing form of core function | Increase acceptability and adoption | Pre; planned, reactive to HS A input | HS clinicians, HIT, QL | HS-wide in HS A only |
| Added a result status for patients who initially accept services then are unreachable to ensure every eReferred patient has a result returned to referring clinician | **Content:** Improved clarity and completeness of eReferral outcome feedback to clinicians | Tweaking; Core functions preserved | Increase acceptability and adoption | Post; planned, reactive to HS input | HS clinicians, HIT, QL | HS-wide |
